# Updating Sacbrood Virus Quantification PCR Method Using a TaqMan-MGB Probe

**DOI:** 10.3390/vetsci8040063

**Published:** 2021-04-13

**Authors:** Wei-Fone Huang, Yakun Zhang, Shahid Mehmood, Zhengwei Wang, Chunsheng Hou, Zhiguo Li

**Affiliations:** 1 College of Animal Science (College of Bee Science), Fujian Agriculture and Forestry University, Fuzhou 350002, China; yakun.zhyk@gmail.com (Y.Z.); zhiguo.li@fafu.edu.cn (Z.L.); 2 Chemical Ecology Group, Key Laboratory of Tropical Forest Ecology, Xishuangbanna Tropical Botanical Garden, Chinese Academy of Sciences, Kunming 650000, China; shahidbee@aup.edu.pk (S.M.); wangzhengwei@xtbg.ac.cn (Z.W.); 3 Institute of Apicultural Research, Chinese Academy of Agricultural Sciences, Beijing 100193, China; houchunsheng@caas.cn; 4 Key Laboratory of Pollinating Insect Biology, Ministry of Agriculture, Beijing 100193, China

**Keywords:** CSBV, AcSBV, Thai SBV, TaqMan, MGB probe

## Abstract

Sacbrood virus (SBV) is a common honey bee virus disease. SBV variants and strains identified in Asian honey bees, *Apis cerana*, have created confusion in identifications. Although the regional names indicated the expansions of the virus in new regions, pathogenesis, and genomes of these variants are not distinct enough to be a separate virus species. However, current SBV qPCR methods may not detect newly identified *A. cerana* SBV variants (Ac SBV) according to the genome sequences. Since these Ac SBV can naturally infect *A. mellifera* and possibly other hymenopterans, ignorance of Ac SBV variants in detection methods is simply unwise. In this report, we updated the qPCR method based on Blanchard’s design that used conserved regions of VP1 to design a TaqMan method with an MGB (minor groove binder) probe. We tested the method in bees and hornets, including *A. mellifera*, *A. cerana*, and *Vespa velutina*. The updated primers and the probe can match published SBV and Ac SBV genomes in databases, and this updated method has reasonable sensitivity and flexibility to be applied as a detection and quantification method before the discovery of variants with more mutated VP1 gene.

## 1. Introduction

Sacbrood virus (SBV) is a common honey bee virus that causes obvious symptoms in larvae. Symptomatic larvae usually die at the late stages near pupation, which can cause serious resource losses to the colonies. Asymptomatic infections can be found in adult worker bees [1] that still performed nursing or foraging duties, even in colonies that have no significant larva losses due to sacbrood disease. Through the infected workers, SBV was transmitted to uninfected larvae [1] and contaminated collected pollen [2]. 

European honey bees, *Apis mellifera*, usually cope with sacbrood disease better than Asian honey bees, *A. cerana*. *A. cerana* populations can decrease dramatically after the introduction of SBV into the regions [3], whereas *A. mellifera* colony losses caused by SBV were rarely reported in the same environment. In addition to the biological dissimilarities between *A. mellifera* and *A. cerana*, SBV variants adapted to *A. cerana* may have contributed to the differences. A Chinese sacbrood virus variant (CSBV) was found in South China in 1972 [4] with observations of severe *A. cerana* colony losses, and related variants were later reported in North and East China, similar but not identical in sequences [5,6]. Bailey described the Thai sacbrood strain [7] that was later found in the regions across Southeast Asia [8], which also resulted in more than 90% mortalities of local *A. cerana* populations. India reported comparable losses of *A. cerana* after the Thai SBV strain introduction [9]. Similar cases were reported in East Asia after accidental introductions of *A. cerana* SBV variants [3]. 

These *A. cerana* SBV variants with various names [9,10,11] have shown the ability to infect *A. mellifera*. Ac SBV has shown the ability to infect and kill *A. mellifera* larvae in early laboratory trials [7], but the field cases of Ac SBV infections in *A. mellifera* were not reported till the last decade. In Shandong [11] and Fujian (unpublished data), Ac SBV has replaced the original SBV strain in *A. mellifera* colonies and became the dominant SBV variant in all domestic honey bees. Ac SBV in Asian hornet, *Vespa velutina*, was also reported [12]. Although Ac SBV still shows the characteristics of an emerging disease that annihilated naïve *A. cerana* populations as it spreads, it has jumped from *A. cerana* to other hosts and became a potential threat to apiculture.

**Figure 1:**
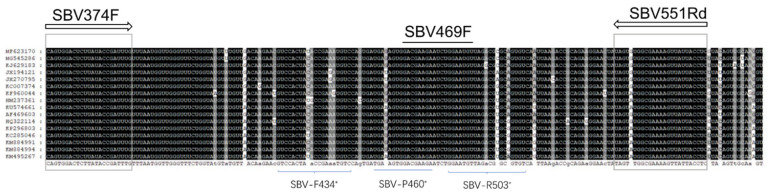
Alignment of the SBV variants. Regions for designing the primers and the probe were annotated on the top. Asterisk labels indicate the primer and the probe locations published by Blanchard et al. [13].

Current quantitative PCR methods may not detect all Ac SBV variants. In recent years, newly published Ac SBV genomes from East and South Asia have quickly expanded the databases due to the expansion of Ac SBV epizootics. According to the variants, mismatched nucleotides in the primers and the probe (Figure 1) were noted in Blanchard’s TaqMan method [13]. Although the Ac SBV genomes published earlier have been considered in the design, a few mismatched nucleotides were allowed in the primers according to their alignment [13]. Another TaqMan method designed for Chinese SBV isolates from North China [14] does not exactly match (two mismatched nucleotides in both the primers and one in the probe) with our Fujian clone and Yunnan Ac SBV collected from East and Southwest China, respectively. Other published methods with SYBR Green qPCR [15] and loop-mediated PCR [16,17] also have a few mismatched nucleotides. These findings surprised us when we attempt to adapt these methods in our studies, and these mismatched nucleotides may lead to false-negative results. Therefore, we updated the SBV TaqMan qPCR method based on Blanchard’s design to make it universally matched the published SBV and Ac SBV genomes in GenBank. The updated primers and the probe have 100% identity with the top 100 SBV sequences found in the database. This method should reliably detect and quantify SBV variants before the discovery of new variants having more mutated VP1 genes. Constantly updating the detection method may be needed for a honey bee virus disease that is still in upsurge.

## 2. Materials and Methods

### 2.1. Sample Collections 

Honey bees collected from Yunan and Fuzhou, Fujian, were included in this study. The Sacbrood virus clones from Fuzhou [18] and Chongqing (Central China) were included. Asian hornet (*Vespa velutina*) samples were collected in Yunan, from reared hornet colonies feeding on the *A. mellifera* colonies that were also collected as testing samples. Bees and hornets at pupa and adult stages were collected. No symptomatic bees were included in this study. The collected samples were kept in RNA keeper (Vazyme, Nanjing, China) and stored at −20 °C according to the manufacturer’s suggestions. Ten *A. cerana* samples, five from Yunnan and five from Fuzhou, 12 *A. mellifera* samples, five from Yunnan and seven from Fuzhou were included in the test. Five *V. velutina* samples from Yunnan were also included to test the applicability of the method in other hymenopterans.

### 2.2. RNA Extraction and Reverse Transcription

RNA extractions were carried out using LabServ Universal RNA Kit (Thermo-Fisher, Waltham, MA, USA) with Kingfisher Flex system (Thermo-Fisher, Waltham, MA) according to the instructions. Extracted RNA samples were reverse transcribed using HiScript^®^ II Reverse Transcriptase with gDNA wiper (Vazyme, Nanjing, China) following the manufacture’s protocol. Produced cDNA samples were diluted to 80 μL, four times the original volume, and stored in −20 °C. The dilution was done to facilitate pipetting the cDNA (1-μL after dilution) into the small reaction volume (5 μL) used in 384-well plates.

### 2.3. Quantification PCR with the TaqMan-MGB (Minor Groove Binder) Probe

The Sacbrood virus VP1 region that was selected in Blanchard et al., 2014 [13] still provides discontinuous conserved regions that were sufficient for designing primers and probes. According to the alignment of SBV genomes, including *A. mellifera* SBV, Thai SBV, Korean isolates, Chinese SBV isolates (from North, East, and South China), we adjusted the regions for primers and stretched the qPCR product to approximately 178bp in size (Figure 1). The forward (SBV374F: CAG TGG ACT CTT ATA CCG ATT TG) and reverse primers (SBV551Rd: GAG GTA ATA ACT TTT CGC CAY ACT A) and the probe (SBV469F: FAM-GAC GAA GAA TCT GGA ATG T-MGB) were designed according to the conserved regions, and the numbers within the names indicated the relative locations within the alignment. The locations were selected and fine-tuned using Beacon Designer™ (Premier Biosoft, San Francisco, CA, USA) and Blastn search results. 

Different primer concentrations and reaction temperatures were first tested using the SYBR Green method, with ChamQ Universal SYBR Mix (Vazyme, Nanjing) in a two-step PCR program before applying to the TaqMan method. 200 nm of the forward and 300 nm of the reverse primer were selected. This combination yielded PCR efficiencies stably within the 90–110% range in the preliminary tests using the SYBR Green method comparing to the equal amount primer of 200 nm. Because a degenerated base was included in the reverse primer, we think the concentration increase is reasonable. We used this combination for the following temperature tests using the gradient temperature function in Bio-rad CFX 384, which was also used as the thermocycler for all qPCR. CFX manager 3.1 (Bio-rad, Hercules, CA, US) was used for the analyses. The TaqMan qPCR used PerfectStart™ II Probe qPCR SuperMix (Transgene), and the reaction temperatures were tested again to find the optimal temperatures in the TaqMan method.

## 3. Results

The updated qPCR primers resulted in sufficient PCR efficiencies (90–110%) within the temperature range of 56–61 °C in the SYBR Green qPCR method; unspecific amplicons, however, were noted in no-template controls (NTC) after 35 cycles. Although melting curve analyses can distinguish the unspecific amplicons (Tm values ranged from 72.5 to 75.5 °C) and the specific amplicon (81.5 °C Tm value), unspecific amplificons can confuse analyses and affect the reliabilities. 

**Figure 2:**
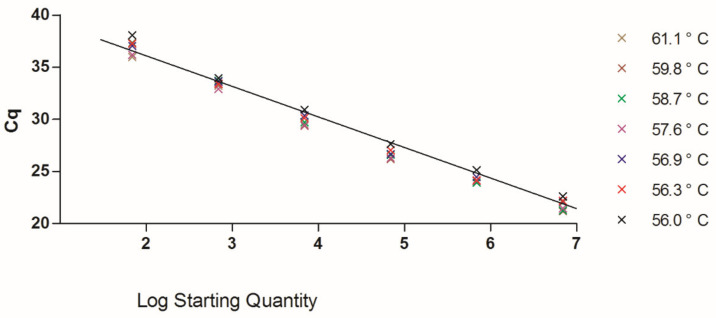
Standard curve generated from SBV clone plasmids (6.9 × 10^6^–6.9 × 10^1^) in gradient temperatures, 56–61 °C, of the second temperature within the 2-step qPCR program. The second temperature setting of the 2-step program influences the accuracy and efficiency of qPCR. Seven temperatures within the range of 56–61 °C, automatically generated by CFX manager 3.1, were tested. The mean Cq values of different temperatures were labeled on the chart. The results showed that all data points generated from different temperature settings have similar Cq values, which indicates all the tested temperatures have acceptable results. The overall PCR efficiency is 111.6%, R^2^ = 0.98.

The updated TaqMan qPCR method used the identical concentrations of the primers in the SYBR Green method. We tested the MGB probe in 250 and 300 nm, and both concentrations generated good PCR efficiencies (90–110%). Both Ac SBV clones from Chongqing and Fuzhou generated strong and low Cq values in the TaqMan qPCR, and we used the Fuzhou clone as standards in the following tests. The reaction temperatures, ranged from 56 °C to 61 °C, also generated PCR efficiencies within the acceptable range (Figure 2). No unspecific amplification was noted in NTCs.

The temperatures higher than 57 °C showed better results when we test the limits of detection (LOD). The LOD of the TaqMan method was between 69 and 6.9 copies (1 × 10^−6^ and 1 × 10^−7^ ng of our clone plasmid), and 6.9 copies generated no signals in some replicate reactions at the reaction temperatures lower than 57 °C. The Cq values of 6.9 copies drifted from 36.26 to 40.52 in replicates (using the same threshold) at different temperatures with high Cq standard deviations (0.61 to 1.26), which is not reliable. These results suggested that the TaqMan can only reliably detect 69 copies in all tested conditions. The overall test results suggested a two-step PCR programming: 95 °C for 3 min preincubation and then followed by 45 cycles of 95 °C for 10 s and 60 °C for 30 s. The last temperature setting has a wide range of temperatures (Figure 2), and it can be adjusted between 57–61 °C. 

Samples collected for another research in Yunnan (unpublished) were randomly selected for testing the updated TaqMan method. *V. velutina* samples did not generate reliable positive results, which is similar to the results that used different SYBR Green qPCR primers [18]. Two *V. velutina* samples have positive results in two of the three repeats, but the numbers were below the reliable LOD. Two *A. mellifera* field samples were positive, from Yunnan and Fuzhou, respectively, and one Yunnan *A. cerana* sample was positive.

## 4. Discussion

Our TaqMan method provides better specificity than the SYBR Green method using identical primers. Although the SYBR green can be as good as the TaqMan method [19], we encountered problems of unspecific amplification using the SYBR Green method with different designs to estimate SBV infections. With the fluorescent-labeled TaqMan probe, the qPCR method showed better specificity and solved the problem. MGB modified TaqMan probe can have short sequences that have lower Tm values than the primers, which allowed us to design probes with ideal properties in the discontinuous, short, and conserved regions in the SBV VP1 gene; the same VP1 gene has been used in Blanchard’s design [13], but they selected a small amplicon, possibly for good sensitivities and PCR efficiencies, that includes some variable regions in the alignment (Figure 1). Interestingly, the SBV VP1 gene has both variable regions that could be associated with adapting different host cells [5] and conserved regions that possibly had structural functions. In the genome alignment using the same data of figure one, more conserved regions were found in the VP1 gene than the RdRp gene that was used for designing another TaqMan assay [14]. Our SYBR Green results using the same primer set were difficult to estimate the LOD because of the unspecific amplification, but the TaqMan showed a LOD between 6.9E1-6.9E0 copies. This LOD may not be excellent for a TaqMan method but can reliably detect a few thousand virions within a bee, which is sensitive enough for application. The flexibility of the reaction temperatures (Figure 2) made the TaqMan qPCR easy to arrange with other house-keeping gene and virus qPCRs at the same PCR plate and reduce the difficulties in adapting the method using a different thermocycler. 

Most of the known honey bee viruses are RNA viruses that have native high mutation rates. As the bee virus epizootics in some areas thrives, new variants and host-switching events should be expected. Reports from East-Asia and India have suggested Ac SBV variants caused epizootics in various regions, sometimes isolated, which makes Ac SBV fits the expectation of new variants and host-switching. Other bee viruses, such as DWV, may fit this expectation as well. Since asymptomatic and latent infections of bee virus diseases are common in the field, the molecular detection tools for these viruses are fundamental and need constant updates to make sure all variants can be monitored efficiently.
